# Chemically Modified Nanoparticles for Enhanced Antioxidant and Antimicrobial Properties with Cinnamon Essential Oil

**DOI:** 10.3390/antiox12122057

**Published:** 2023-11-29

**Authors:** Aaron A. López-Cano, Verónica Martínez-Aguilar, Mariana G. Peña-Juárez, Ricardo López-Esparza, Enrique Delgado-Alvarado, Emmanuel J. Gutiérrez-Castañeda, Mayra Del Angel-Monroy, Elías Pérez, Agustín L. Herrera-May, J. Amir Gonzalez-Calderon

**Affiliations:** 1 Facultad de Ciencias, Universidad Autónoma de San Luis Potosí, San Luis Potosí 78290, San Luis Potosí, Mexico; a296339@alumnos.uaslp.mx; 2 Doctorado Institucional en Ingeniería y Ciencia de Materiales, Universidad Autónoma de San Luis Potosí, San Luis Potosí 78210, San Luis Potosí, Mexico; a332908@alumnos.uaslp.mx; 3 Departamento de Ciencias Básicas, Tecnológico Nacional de México, Instituto Tecnológico de Orizaba, Orizaba 94320, Veracruz, Mexico; mariana.pj@orizaba.tecnm.mx; 4 Departamento de Física, 1626, Universidad de Sonora, Hermosillo 83000, Sonora, Mexico; ricardo.lopez@unison.mx; 5 Micro and Nanotechnology Research Center, Universidad Veracruzana, Boca del Río 94294, Veracruz, Mexico; endelgado@uv.mx; 6 Cátedras CONAHCYT-Instituto de Metalurgia, Universidad Autónoma de San Luis Potosí, San Luis Potosí 78210, San Luis Potosí, Mexico; emmanuel.gutierrez@uaslp.mx; 7 Especialidad en Plásticos y Materiales Avanzados, CIATEQ A.C., San Luis Potosí 78395, San Luis Potosí, Mexico; mayra.delangel@ciateq.mx; 8 Instituto de Física, Universidad Autónoma de San Luis Potosí, San Luis Potosí 78000, San Luis Potosí, Mexico; elias.perez@uaslp.mx; 9 Maestría en Ingeniería Aplicada, Facultad de Ingeniería de la Construcción y el Hábitat, Universidad Veracruzana, Boca del Río 94294, Veracruz, Mexico; 10 Cátedras CONAHCYT—Instituto de Física, Universidad Autónoma de San Luis Potosí, San Luis Potosí 78290, San Luis Potosí, Mexico

**Keywords:** cinnamon essential oil, antioxidant activity, antimicrobial properties, nanoparticles, polylactic acid films

## Abstract

We explored the potential of different nanoparticles (TiO_2_, CaCO_3_, and Al_2_O_3_), considering their pure form and modified with cinnamon essential oil (CEO). These materials were characterized using various techniques, including FTIR spectroscopy, XRD analysis, TGA, and SEM. The interaction between CEO and nanoparticles changed depending on the nanoparticle type. Al_2_O_3_ nanoparticles exhibited the strongest interaction with CEO, increasing their antioxidant capacity by around 40% and their transfer of antimicrobial properties, particularly against Gram-negative bacteria. In contrast, TiO_2_ and CaCO_3_ nanoparticles showed limited interaction with CEO, resulting in lower antioxidant capacity and antimicrobial activity. Incorporating pure and CEO-modified nanoparticles into polylactic acid (PLA) films improved their mechanical and thermal properties, which are suitable for applications requiring greater strength. This research highlights the potential of metal oxide nanoparticles to enhance the antimicrobial and antioxidant capabilities of polymers. In addition, incorporating cinnamon essential oil can increase the antioxidant and antimicrobial effectiveness of the metal oxide nanoparticles and improve the mechanical and thermal properties of PLA films. Thus, these PLA films exhibit favorable characteristics for active packaging applications.

## 1. Introduction

In recent studies, inorganic chemical compounds have played a significant role in metallurgy and materials science research. Due to their complexity, researchers have continually enhanced their chemical and physical properties, introducing new qualities that find applications across multiple disciplines. Some of the most utilized include Copper (II) oxide (CuO), Titanium dioxide (TiO_2_), Aluminum oxide (Al_2_O_3_), and Calcium carbonate (CaCO_3_), to name a few. These properties are further enhanced when these materials are manipulated at nanoscale dimensions, providing unique electrical, optical, catalytic, magnetic, and mechanical properties. These compounds find applications in various fields, including gas sensors, fuel cells, advanced ceramics, chemical and biosensors, batteries, solar cells, piezoelectric, super-capacitors, catalysts, anticorrosion coatings, and more [1]. However, one of the most extensively studied applications in recent years is their incorporation into polymeric matrices, where several studies have reported improvements primarily in mechanical and thermal properties. For example, TiO_2_ is a versatile material extensively used in polymeric matrices which can enhance the properties of polymers in numerous ways. The addition of these nanoparticles to polymers can enhance their mechanical properties. This includes increased tensile strength, stiffness, and abrasion resistance, making them suitable for applications requiring durable materials. In addition, incorporating TiO_2_ in the polymeric matrices can improve their thermal stability, making them suitable for high-temperature applications. Also, some forms of TiO_2_ nanoparticles have demonstrated antimicrobial properties.

Al_2_O_3_ is a valuable additive incorporated into polymeric matrices, contributing to improved properties and performance characteristics. Al_2_O_3_ enhances polymers’ mechanical strength and durability, making them suitable for applications requiring high tensile strength and abrasion resistance. Its excellent thermal stability extends the usability of polymers in high-temperature environments. Furthermore, Al_2_O_3_ acts as a reinforcement agent, improving polymeric materials’ stiffness and dimensional stability. Also, alumina is biocompatible, making it suitable for medical applications such as dental implants and orthopedic prostheses.

On the other hand, CaCO_3_ incorporated into polymers can improve their mechanical strength and stiffness. This reinforcement is especially valuable in applications where materials need to withstand mechanical stress and structural loads, such as in automotive parts, construction materials, and packaging. CaCO_3_ can enhance the thermal stability of polymers, allowing them to withstand higher temperatures without deformation or degradation. As a naturally occurring mineral, CaCO_3_ is considered an environmentally friendly filler. Its use in polymers can contribute to the development of more sustainable materials.

Poly(lactic) acid (PLA) is a biodegradable polymer with excellent properties and it has been a subject of study for enhancing its polymeric matrix by incorporating these previous compounds. Three primary methods for achieving this modification are in situ polymerization of monomers in the presence of metal oxide nanoparticles, direct mixing of nanoparticles in solution, or via the sol-gel process. Since 2002, research has been ongoing in the application of metal oxide nanoparticles in polymers. However, active or intelligent packaging has gained traction due to the increasing demand for developing new technologies.

Active packaging contains an active ingredient capable of releasing components that inhibit microbial growth and oxidation within its containment barrier, improving the stability of the packaged product typically used in the food industry. Active ingredients can be synthetic or natural preservatives. For instance, essential oils exhibit antifungal, insecticidal, antiviral, antiseptic, and disinfectant properties. Essential oils consist of various functional groups in their structures, including alcohols, ketones, aldehydes, and lactones in the terpenoids present in essential oils. These active functional groups at different positions can function as both reducers and stabilizers. Thus, more investigations on essential oils incorporated with metal oxide nanoparticles are required. Terpenoids in essential oils can be absorbed through interactions involving pi (π)-electrons or carbonyl groups and other strong chelating agents [2].

In this work, we evaluated the chemical modification of TiO_2_, CaCO_3_, and Al_2_O_3_ nanoparticles with cinnamon essential oil (CEO) to study its effects on the chemical, physical, and thermal properties of the nanoparticles. Furthermore, we assessed the mechanical and thermal properties of PLA films that incorporated pure and CEO-modified nanoparticles. The experimental results showed that the CEO-modified Al_2_O_3_ nanoparticles improved their antioxidant capability and antimicrobial properties. Also, the CEO-modified nanoparticles added to PLA films increased their mechanical and thermal properties. Thus, these enhanced PLA films could be employed in active packaging in the food industry.

## 2. Materials and Methods

### 2.1. Fabrication

#### 2.1.1. Materials

Rutile phase titanium dioxide (TiO_2_) with a purity of 99% and a particle size of 20 nm and aluminum dioxide (Al_2_O_3_) with a purity of 99% and a particle size of 20 nm were acquired through IoLiTec. Calcium carbonate (CaCO_3_) was obtained from Xiamen Haichuanda Industry and Trade Co., Ltd., presenting a purity of 94% and an average particle size of 15–40 nm. Ethanol (CTR, Scientific, Monterrey, Mexico, purity 99.95%) was employed as the solvent. Cinnamomum verum essential oil (CEO) was obtained from lifeScent (purity 99.99%). PLA (Polylactic Acid) designed for 3D printing was used to prepare the films.

#### 2.1.2. Nanoparticle Modification

The methodology followed was based on impregnating 200 µL of Cinnamomum verum essential oil per gram of nanoparticles. Subsequently, 5 mL of ethanol was added to facilitate agitation, which was carried out for 24 h to ensure uniform particle dispersion in the oil. Before transferring the CEO into the container, it was covered with aluminum foil to prevent photosensitivity. After the 24 h period, the nanoparticles were dried for 24 h at 50 °C.

#### 2.1.3. Film Fabrication

Film fabrication was performed using the casting method. Initially, 1.5 g of PLA was weighed and dissolved in 20 mL of chloroform, and magnetic stirring was maintained for approximately 1 h until a homogeneous mixture was achieved. Subsequently, nanoparticles were added at a concentration of 0.1% *w*/*w* with respect to PLA. The mixture was stirred for 40 min, followed by 20 min of sonication to eliminate bubbles and ensure proper nanoparticle dispersion on the film. Finally, the mixture was poured into Petri dishes and allowed to dry at room temperature.

### 2.2. Characterization of Nanoparticles

#### 2.2.1. Fourier-Transform Infrared Spectroscopy (FTIR)

The FTIR spectra of the nanoparticles were determined using an Agilent Cary 660 spectrometer in attenuated total reflectance (ATR) mode in the range of 4000–400 cm^−1^. The nanoparticles were placed on the ATR sample holder, and the instrument was operated to record the spectra.

#### 2.2.2. X-ray Diffraction (XRD)

For XRD analysis, nanoparticle samples were placed in a sample holder of the Malvern Panalytical Empyrean instrument, using a Cu Kα radiation source filtered with nickel, with an angular range of 8–80° at a 2θ step and a voltage of 40 kV.

#### 2.2.3. Thermogravimetric Analysis (TGA)

Thermogravimetric analysis (TGA) of the samples was carried out using an SDT 650 model TA Instruments simultaneous thermal analyzer. The nanoparticle samples for TGA were placed in an aluminum cell at quantities ranging from 0 to 15 µg. The samples were analyzed using a heating rate of 10 °C/min and temperature range from 25 to 600 °C under a nitrogen atmosphere.

#### 2.2.4. X-ray Photoelectron Spectroscopy (XPS)

In pursuit of elucidating the molecular bonding mechanism between cinnamon essential oil (CEO) molecules and the nanoparticle surface, an X-ray photoelectron spectroscopy (XPS) analysis was conducted on the most promising system. This analysis can discern the chemical composition of surface structures and ascertain the chemical state of their elemental components. Employing a Perkin Elmer PHI5100 system within an ultrahigh vacuum (UHV) environment facilitated the XPS analysis. A dual-source X-ray from MgKα (hν = 1256 eV) at 300 W with a 15.0 kV polarized anode was used. High-resolution spectra were recorded with a constant energy step (CAE) E0 = 22.36 eV, while the full-scan spectrum utilized E0 = 71.55 eV, angled at 54° to the surface normal. Energy position calibration hinged on the Ag 3d5/2 orbital at 368.20 eV, offering a resolution (FWHM) of 1.10 eV, with references to Au 4f7/2 at 84.00 eV and C 1s at 284.75 eV.

#### 2.2.5. Antioxidant Activity

The antioxidant activity of the modified particles was determined using the 2,2-Diphenyl-1-picrylhydrazyl (DPPH) radical assay [3,4]. Different concentrations of each antioxidant (expressed as moles of antioxidant/mole of DPPH) were tested, with final concentrations of 4, 8, 12, 16, and 20 mg/mL. A solution of DPPH in ethanol at a concentration of 6 × 10^−5^ mol/L was prepared. An aliquot of 3.9 mL of the DPPH solution was mixed with 0.1 mL of the samples at various concentrations and agitated. After 30 min of incubation at room temperature in the dark, absorbance values (*Abs*) were measured at 517 nm using a UV-vis spectrophotometer, and the inhibition percentage was calculated as shown in Equation (1).

(1) $$Inhibition\;(\%)=\frac{{Abs}_{control}-{Abs}_{sample}}{{Abs}_{control}}\times 100$$

#### 2.2.6. Antimicrobial Activity

The CLSI standardized method involving microdilution and drop counting was employed to assess the antimicrobial activity of the nanoparticles. *Escherichia coli* (ATCC 25922) and *Enterococcus faecalis* (ATCC 29212) were selected as target bacteria for the analysis. These bacteria were cultured on soy agar medium and identified using the Gram stain method. The chosen CLSI method provides a reliable and recognized approach for evaluating the antimicrobial properties of substances against specific bacterial strains. A 200 mg/mL stock solution was serially diluted to 100, 50, and 25 mg/mL. Muller Hilton broth and ultrapure water were utilized for preparing the microdilutions. Inoculated microplates were incubated at 34 °C and 90 rpm for 24 h. Subsequently, 20 µL of each well was diluted five times in 180 µL of Muller Hilton broth. Finally, 10 µL of each dilution was plated on Trypticase Soy Agar to observe bacterial growth and potential inhibition by counting the number of colonies. The formula in Equation (2) was applied for a more precise result.

(2) $$CFU/mL=\frac{number\;of\;colonies\;counted\times dilution\;factor}{mL\;of\;inoculated\;sample}$$

### 2.3. Characterization of Films

#### 2.3.1. Fourier-Transform Infrared Spectroscopy (FTIR)

For FTIR analysis on the films, the same spectrometer as in Section 2.2.1 was used, along with the same conditions. The film samples were cut into 2 × 2 cm pieces and placed on the ATR sample holder of the instrument.

#### 2.3.2. X-ray Diffraction (XRD)

The films were placed on a sample holder of the XRD equipment to analyze their X-ray diffraction. The films were previously cut into small squares of 0.5 × 0.5 cm and placed on graphite pins. Then, they were coated with a conductive material (silver) before being measured.

#### 2.3.3. Scanning Electron Microscopy (SEM)

The surface morphology of the films was examined using high-resolution scanning electron microscopy (SEM) (XL30-SFEG, Philips/FEI, Hillsboro, OR, USA). Before examination, the films were coated with a thin layer of gold and inspected at an acceleration voltage of 20 kV.

#### 2.3.4. Differential Scanning Calorimetry (DSC)

Differential scanning calorimetry analysis was conducted using a Perkin Elmer model 8500 DSC. The sample was placed in a sealed aluminum capsule containing between 6 and 15 mg. The samples were scanned at a rate of 10 °C/min in a temperature range of 25–200 °C. The glass transition temperature (Tg), crystallization temperature (Tc), and melting temperature (Tm) were obtained. The degree of crystallinity (*Xc*) was calculated using Equation (3):

(3) $$Xc\left(\%\right)=\left(\frac{{∆H}_{m}-{∆H}_{c}}{{∆H{}^{\circ}}_{m}}\right)*100$$

where ${∆H}_{m}$ represents the enthalpy of fusion, ${∆H}_{c}$ is the enthalpy of crystallization, and ${∆H{}^{\circ}}_{m}$ is the heat of fusion for 100% crystallinity of the PLA homopolymer, which is 93.7 J/g.

#### 2.3.5. Water Vapor Permeability (WVP)

WVP was determined by quantifying the rate of water vapor movement through the film by measuring weight changes due to moisture transfer. The desiccant method of the gravimetric technique (ASTM E-96-95 [5]) was chosen. Duplicate tests were performed using 5.4 cm diameter Petri dishes, with 10 mL of a NaCl and CaCl_2_ solution at a concentration of 142 mmol/liter of sodium ions and 2.5 mmol/liter of calcium ions placed beneath the films. The Petri dishes were initially weighed with the content and then with the film. They were placed in a desiccator with silica gel at room temperature for 6 h. Weight measurements were taken every hour during the designated time. Equations (4) and (5) were used for calculations, where *WVTR* is the water vapor transmission rate (*Δm/Δt*), *A* is the exposed area of the film, *WVP* is the water vapor permeability, *X* is the film thickness, P is the vapor pressure of saturated water vapor (Pa) at the test temperature (25 °C), *R*1 is the relative humidity in the desiccator, and R2 is the relative humidity in the cup.

(4) $$WVTR=\frac{∆m}{∆t*A}$$

(5) $$WVP=\frac{WVTR}{P\left(R2-R1\right)}X$$

### 2.4. Mechanical Properties

The mechanical properties of the films, including tensile strength (TS), elongation at break (EB), and Young’s modulus (YM), were estimated following the ASTM standard method D882-18 [6] with some modifications. A texture analyzer (Texture Pro CT Build 35, Brookfield, WI, USA) was used to measure the mechanical properties at a cross-head speed of 1 mm/s and an initial grip of 15 mm. Tensile strength (MPa) was calculated by relating the maximum load to the initial cross-sectional area of the sample. Elongation at break (%) was determined as the percentage change in length from the initial 50 mm to the point of sample failure. Young’s modulus (MPa) is the stress-to-strain ratio and is a measure of film stiffness, as shown in Equations (6)–(8).

(6) $$TS=\frac{Stress\;at\;breaking\;point}{Film\;thickness*film\;width}$$

(7) $$\%EB=\frac{Increase\;of\;the\;film\;length}{Initial\;film\;length}\times 100$$

(8) $$YM=\frac{Longitudinal\;stress}{Longitudinal\;deformation}$$

## 3. Results and Discussion

### 3.1. Characterization of Nanoparticles

#### 3.1.1. FTIR

**Figure 1:**
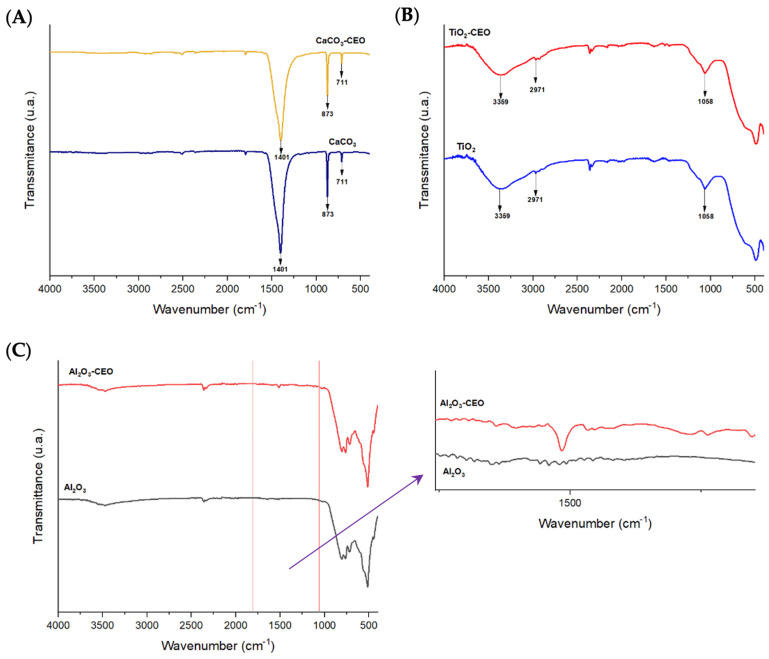
FTIR spectra in nanoparticles of (**A**) CaCO_3_, (**B**) TiO_2_, and (**C**) Al_2_O_3_.

Infrared spectroscopy is a crucial qualitative analysis technique for determining molecules present in a sample. In this context, FTIR was used to confirm the presence of cinnamon essential oil in the metal oxide nanoparticles. Figure 1 displays the infrared spectra obtained for each nanoparticle with and without chemical modification using essential oil. In Figure 1A, the CaCO_3_ spectrum exhibits characteristic bands at a wavelength of 1401 cm^−1^, corresponding to the asymmetric stretching vibration of the O-C-O functional group, 873 cm^−1^ represents the bending vibration mode of the same group, and 711 cm^−1^ indicates the out-of-plane vibration of the O-C-O group [7,8]. Tizo et al. [9] reported a similar spectrum obtained from eggshells, revealing bands at 712 and 876 cm^−1^ attributed to C-H bending vibration, along with a prominent band at 1426 cm^−1^ corresponding to the stretching vibration of carbonate groups. Kerru et al. (2020) [10] also observed a similar pattern, emphasizing the significant absorption band associated with the stretching vibrations of C-O and O-C-O bonds located at 1407 and 889 cm^−1^. In another study, Dehghani et al. [11] presented the FTIR spectrum of calcium carbonate nanoparticles synthesized by the precipitation method. In their findings, the bands at 712, 848, and 872 cm^−1^ were attributed to the CO_3_ (calcite) vibrational mode of calcium carbonate nanoparticles, while the band at 1406 cm^−1^ was associated with the symmetric stretching vibration of CO_3_.

In Figure 1B, the TiO_2_ spectrum shows the main bands of this molecule at 3359 cm^−1^, corresponding to symmetric and asymmetric stretching vibrations of the hydroxyl group (Ti-OH), and the band at 2971 cm^−1^ could be attributed to the vibrational stretching mode of the C-H group [12]. The mentioned oxides did not exhibit any characteristic bands of CEO, indicating that there is no strong interaction between the nanoparticles and CEO. On the other hand, the infrared spectrum of Al_2_O_3_ shown in Figure 1C reveals an extended band at 510 cm^−1^, which could be attributed to the bending vibration of the O-Al-O bond. Another less intense band is observable at 2354 cm^−1^ corresponding to the stretching vibration of the C-H functional group. Between the 1000 to 2000 cm^−1^ range, we observed a band at 1519 cm^−1^ in the nanoparticles loaded with CEO. According to the literature, this wavelength corresponds to the characteristic cinnamaldehyde functional group of cinnamon essential oil [13]. Zhou et al. [14] reported a similar band observed at the wavelength of 1575 cm^−1^, corresponding to the vibration of the benzene ring of cinnamon essential oil (CEO). Similarly, Han et al. [15] identified this band as characteristic of an aromatic ring present in the functional group of CEO, along with other bands at 1624 cm^−1^ corresponding to the vibration of the carbonyl group C=O. This could be attributed to the high proportions of cinnamaldehyde and aldehydes in CEO. Shao et al. [16] observed a similar spectrum in their research on microcapsule samples loaded with cinnamon essential oil. Likewise, Ahmed et al. [17] identified the presence of a band at 1518 cm^−1^ and between 1300 to 800 cm^−1^, attributed to the bending of the C-H bonds of the aromatic ring, confirming the interaction between CEO and low-density polyethylene films. These findings collectively suggest a strong bond between Al_2_O_3_ and CEO.

#### 3.1.2. XRD (X-ray Diffraction)

**Figure 2:**
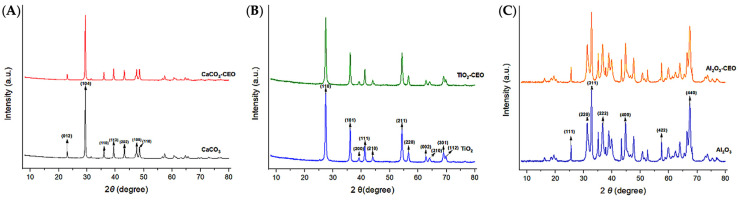
XRD in nanoparticles of (**A**) CaCO_3_, **(B**) TiO_2_, and (**C**) Al_2_O_3_.

X-ray diffraction is a qualitative and quantitative technique that allows us to obtain information about the internal structure of a sample, as well as possible impurities and their degree of crystallinity. Metal oxide nanoparticles were analyzed using this technique to observe potential modifications in the chemical structure when interacting with cinnamon essential oil. Figure 2 presents the obtained diffractograms for each pure sample and those with essential oil.

For CaCO_3_ nanoparticles, as shown in Figure 2A, we can visualize the characteristic crystallographic planes (012), (104), (110), (113), (202), (018), and (116) [18,19]. These crystallographic planes have been observed by other authors. For instance, Render et al. [20] obtained nanoparticles of 15 to 30 nm of CaCO_3_ from eggshells for enteric drug delivery. Boudaria et al. [21] observed a semi-crystalline spectrum in CaCO_3_ nanoparticles of 10 to 20 nm, which differs from the one obtained in this study. However, similar crystallographic planes are present due to their calcite crystalline form. Also, Kirboga et al. [22] and Ferreira et al. [23] observed similar crystallographic planes in CaCO_3_, indicating a rhombohedral morphology. It is possible to determine that cinnamon essential oil is not causing alterations or modifications in the crystalline structure of CaCO_3_. Similarly, in Figure 2B, the diffractogram of TiO_2_ reveals the crystallographic planes (110), (101), (200), (111), (210), (211), (220), (002), (310), (301), and (112) [24,25]. This is consistent with the results of Joni et al. [26], who worked with TiO_2_ nanoparticles in the rutile phase and reported positions at 2***θ*** of 27.42°, 36.08°, 41.25°, 54.33°, and 63.44°, corresponding to the crystallographic planes mentioned in this article. Additionally, Jalali et al. (2020) [27] developed rutile phase TiO_2_ nanoparticles using TiO(OH)_2_ as a precursor for biosynthesis. Saravanan et al. [28] informed the same crystallographic planes, matching the JCPDS card 04-0551 for rutile phase TiO_2_.

The diffractogram of Al_2_O_3_ (Figure 2C) exhibits an amorphous structure with less extended peaks, indicating lower crystallinity. It displays the following crystallographic directions (111), (220), (311), (222), (400), and (440) [29,30]. This spectrum closely resembles that reported by Kim et al. [31], who mention that Al_2_O_3_ nanoparticles are structurally complex oxides and can exist in various metastable phases such as gamma, theta, and alpha. The most representative peaks found at 2***θ*** in their study are 25.57°, 35.14°, 37.76°, 43.33°, 46.16°, 52.53°, 57.47°, 61.27°, 66.49°, 68.18°, and 76.84°, which are attributed to the rhombohedral structure. Similarly, Mohammed et al. [32] confirmed peaks at 43.8°, 35.0°, 57.4°, 25.4°, 68.1°, 52.5°, 37.7°, and 66.5°, indicating that the nanoparticles have a polycrystalline and rhombohedral structure. It is evident that the interaction of cinnamon essential oil with the studied nanoparticles does not generate significant changes in the molecular structure and does not reduce their crystallinity. Until now, no research has been found that demonstrates a similar effect on metal oxide nanoparticles with essential oils.

#### 3.1.3. Thermogravimetric Analysis (TGA)

**Figure 3:**
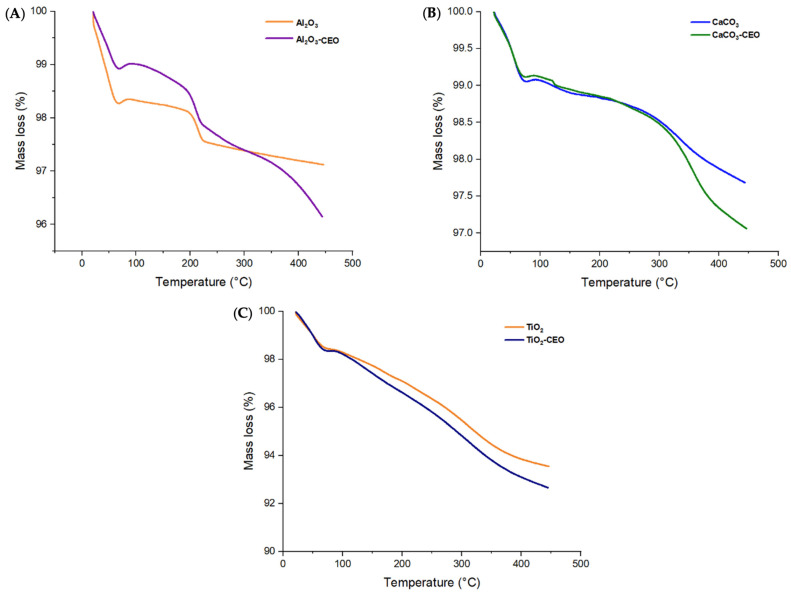
TGA in nanoparticles of (**A**) Al_2_O_3_, (**B**) CaCO_3_, and (**C**) TiO_2_.

Thermogravimetric analysis provides information about the thermal stability of a sample and its potential changes with temperature, including mass loss. Figure 3 displays the thermograms obtained for the metal oxide nanoparticles with and without CEO in a temperature range from 25 °C to 400 °C.

Pure Al_2_O_3_ exhibits its first mass loss (1.74%) at 69 °C (Figure 3A), corresponding to the release of volatile material and possibly absorbed moisture in the nanoparticles. Subsequently, the second mass loss (0.04%) occurs in the temperature range of 67 to 200 °C, and the third loss (0.56%) spans from 200 to 222 °C. In contrast, when loaded with CEO, we can observe the same temperature ranges for the losses. However, a fourth mass loss is noticeable in the range of 222 to 370 °C, indicating that the interaction of CEO with Al_2_O_3_ increases intermolecular forces, thereby enhancing the thermal stability of pure Al_2_O_3_. Moreover, the mass loss is lower, resulting in values of 1.09%, 0.41%, 0.63%, and 0.86% for the first, second, third, and fourth losses, respectively. Du et al. [33] confirmed that Al_2_O_3_ particles had a low mass loss percentage of less than 1%, and when modified with polydopamine, they had a loss of 7.01%. This suggests that cinnamon essential oil adheres better to Al_2_O_3_ than polydopamine. Bristy et al. [34] reported a higher mass loss in pure γ-Al_2_O_3_ particles from 90% to 50%. In addition, when modified with Fe_3_O_4_, the loss is slightly reduced, indicating a good interaction between these components. The Al_2_O_3_ used in this research work is α-Al_2_O_3_, which exhibits better thermal stability.

Pure CaCO_3_ shows an initial mass loss between 25 °C to 76 °C (Figure 3B) equivalent to 0.88%, which corresponds to the loss of volatile material. Subsequently, the curve exhibits a second mass loss (0.05%) around 76 °C to 120 °C and finally another mass loss (0.23%) from 120 °C to 216 °C. In contrast, the nanoparticles modified with CEO show similar first and second mass losses in the same temperature range. The first mass loss is 0.95%, and the second mass loss is 0.22%. However, the final mass loss (0.18%) occurs in the range of 200 to 271 °C, indicating an increased thermal stability of pure CaCO_3_. Siva et al. [35] studied the thermogram of CaCO_3_ in its three phases (calcite, aragonite, and vaterite), in which the calcite registered the highest thermal stability, registering a considerable loss between 600 to 800 °C. On the other hand, we only measured the temperature range up to 400 °C, reporting a similar behavior. At 400 °C, we did not observe a strong mass loss. This agrees with the results of CaCO_3_ in the calcite phase measured by Wang et al. [36].

Pure TiO_2_ nanoparticles (Figure 3C) only display two mass losses of 0.55% and 2.67% from 25.73 °C to 73 °C and 75.73 °C to 281 °C, respectively. The behavior of nanoparticles loaded with CEO is very similar to that of pure TiO_2_, with a higher percentage of mass loss due to the CEO. This contrasts the results of Asir et al. [37], in which the thermogram obtained for TiO_2_ particles in the anatase phase had less thermal stability than those of rutile. In their research, the anatase phase particles began their degradation transition between 150 and 200 °C. When doped with nitrogen, it improved their thermal stability, indicating a better interaction. Tsega et al. [38] obtained a TGA curve similar to the one reported in this article. In their study, the first degradation curve for rutile phase TiO_2_ occurred before 100 °C and continued decreasing until 400 °C. Increasing the concentration of the precursor, Tetra n-butyl orthotitanate, had no effect on the nanoparticles’ thermal stability, a behavior similar to that observed with cinnamon essential oil, which had no favorable effects on TiO_2_.

#### 3.1.4. X-ray Photoelectron Spectroscopy (XPS)

**Figure 4:**
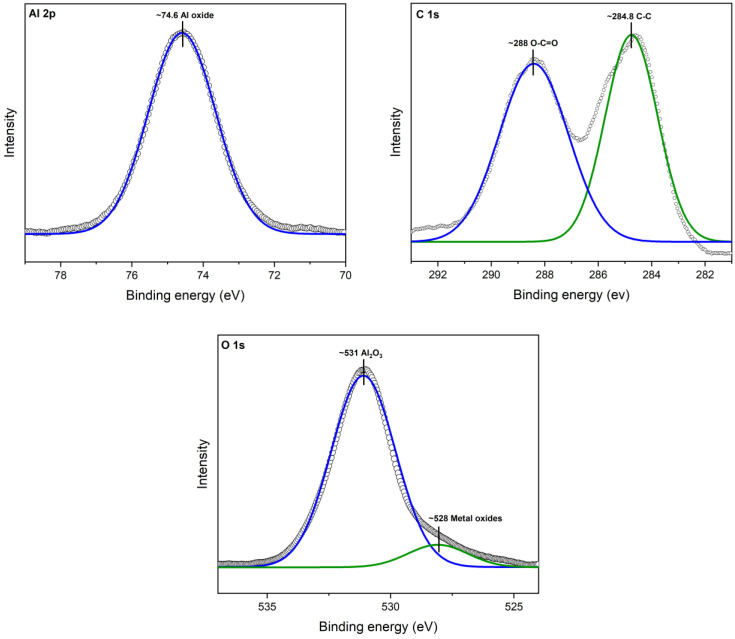
XPS spectra for Al_2_O_3_.

XPS analysis revealed the interaction between aluminum (Al), oxygen (O), and carbon (C) atoms, shedding light on the strong bond formed between cinnamon essential oil (CEO) and Al_2_O_3_ nanoparticles. A deconvolution of the raw XPS spectra depicted the presence of elemental species. Figure 4 illustrates the XPS results for the Al 2p core level, situated at 74.6 eV. Additionally, adventitious C 1s and a C-C peak at 284.8 V were identified. Oxygen was observed as O 1s at 531 eV, confirming the presence of the Al_2_O_3_ chemical state.

**Figure 5:**
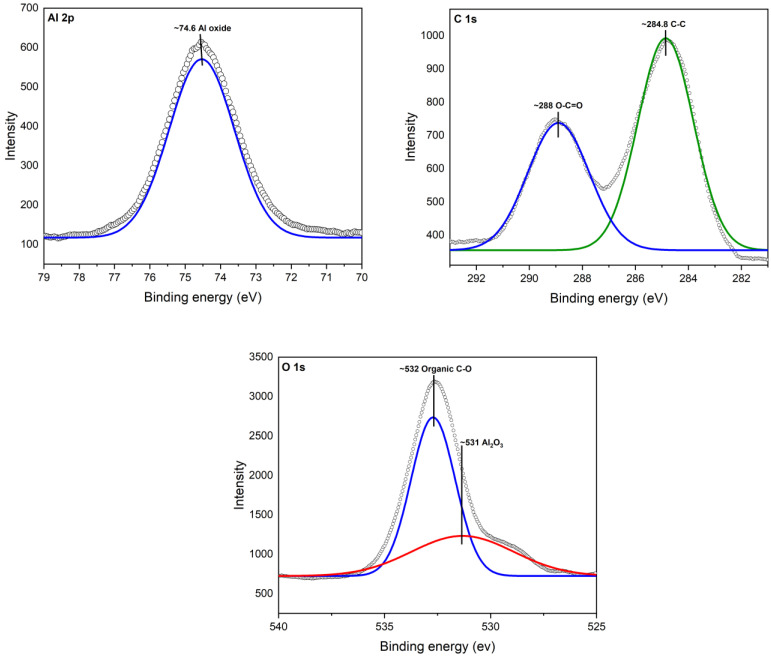
XPS spectra for Al_2_O_3_-CEO.

In Figure 5, the XPS spectra of Al_2_O_3_ after modification with CEO are illustrated. Notably, a new peak appeared for O 1s at ~532, corresponding to the chemical species of organic C-O. This observation confirms the presence of CEO on the Al_2_O_3_ surface, where it is now chemically bonded, primarily due to interactions with oxygen atoms. It is worth mentioning that the observed peaks were compared with Thermo Scientific’s Advantage data system for XPS [39].

This XPS analysis validates the chemical composition and surface modification of the nanoparticles, providing insights into the mechanism behind the enhanced properties of the Al_2_O_3_-CEO system. 

#### 3.1.5. Antioxidant Activity

**Figure 6:**
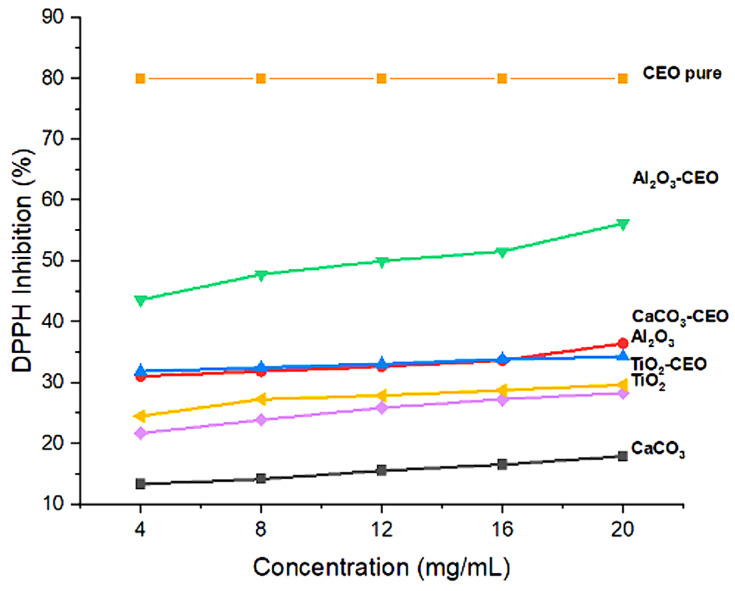
Antioxidant activity in nanoparticles.

Essential oils can inhibit free radicals, which is a characteristic of great interest to our research. In this context, pure nanoparticles and nanoparticles loaded with CEO were evaluated for their antioxidant activity to determine if this property could be transferred to the metal oxide nanoparticles. Figure 6 shows the results of the inhibition degree of these nanoparticles. Among them, Al_2_O_3_ loaded with CEO exhibited a high inhibition value of 55%, followed by CaCO_3_ with CEO at 35% and TiO_2_ with CEO at 28%. Pure CEO demonstrates an 80% inhibition of the DPPH radical [40], indicating that approximately 30% of this inhibition is captured by the Al_2_O_3_ nanoparticles due to a strong interaction between them. In contrast, the pure nanoparticles exhibited low inhibition percentages, suggesting that this evaluated property can be transferred to the nanoparticles with a higher concentration, resulting in a better inhibitory effect. There is limited research on the interaction of metal oxide nanoparticles with cinnamon essential oil, restricting our ability to make direct comparisons with other research works. However, some researchers have reported the antioxidant activity of cinnamon essential oil (CEO). For instance, Liu et al. [41] described a lower percentage of inhibition than that obtained in the present work at a concentration of 10 mg/mL, with a value of 40%, while achieving a value of 82% inhibition at a higher concentration of 80 mg/mL. Similarly, in the research of Chen et al. [42], values comparable to those obtained for CEO were registered. At concentrations below 10 mg/mL, they maintained between 85–90% inhibition, reaching 100% inhibition at a concentration of 20 mg/mL. Also, this research mentioned that the antioxidant activity of CEO is similar to that of clove essential oil and lower than that of thyme, mint, and lavender essential oils.

#### 3.1.6. Antimicrobial Evaluation

**Figure 7:**
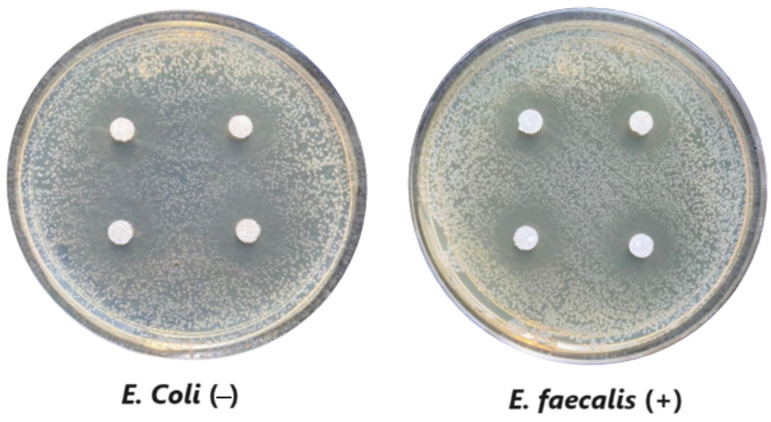
Inhibitory effect of CEO.

The microdilution and drop count methods were employed to assess the microbiological inhibition of nanoparticles loaded with cinnamon essential oil (CEO) and those without it. A disk diffusion test was initially conducted for pure CEO. In Figure 7, a clear inhibition zone of 12 mm (0.6 cm ratio) against both used bacteria (*Escherichia coli* and *Enterococcus faecalis*) demonstrates its antimicrobial effect. Kaskatepe et al. [43] determined the inhibition halo for cinnamon essential oil, reporting a high inhibition value for *E. faecalis* at 28 mm and for *E. coli* at 33 mm. These values are considerably higher than those obtained in our study. They suggest that the inhibitory effect could be correlated with the percentage content of cinnamaldehyde. Similarly, Atki et al. [44] investigated the antimicrobial activity of cinnamon essential oil using the Disc Diffusion Assay, where they obtained a notable value in the inhibition zone, reaching 29 mm for *E. coli*. On the other hand, Parisa et al. [45] observed a lower inhibition halo for cinnamon essential oil compared to our research with a measurement of 9.63 mm at a concentration of 40% for *E. coli*.

**Figure 8:**
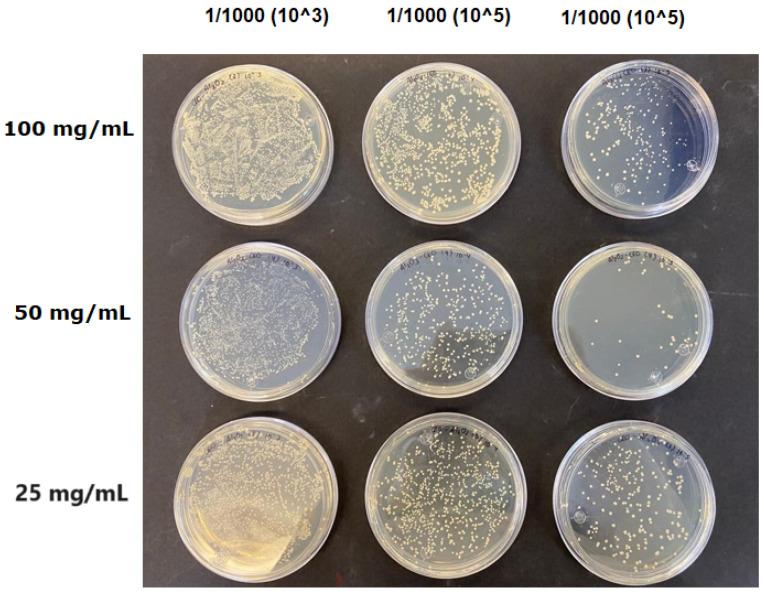
Al_2_O_3_-CEO nanoparticles at different concentrations.

The results obtained with nanoparticles indicate a reduced effect of CEO on them. Table 1 summarizes the inhibitory effect for each nanoparticle concentration used. Pure TiO_2_ and CaCO_3_ nanoparticles and those containing CEO did not exhibit such inhibitory effects on their own, whether with or without CEO. Sunaryono et al. [46] synthesized TiO_2_ nanoparticles and assessed their antibacterial activity using the disc diffusion test. They found that pure TiO_2_ exhibited a zone of inhibition of 8.94 mm for *E. coli* bacteria and 6.72 mm for Gram-positive bacteria such as *S. aureus*. Despite having some inhibitory effect, it was relatively low, aligning with our research, as TiO_2_ alone does not demonstrate strong antimicrobial efficacy. In contrast, Al-Azzawi et al. [47] studied the antimicrobial activity of pure CaCO_3_ nanoparticles, achieving sensitivity to inhibition in *E. coli* bacteria at higher concentrations, with a reported MIC of 200 µg/mL. This result contrasts with our results, where an increase in concentration, coupled with the addition of CEO, did not lead to a significant difference in bacterial inhibition. However, when evaluating Al_2_O_3_ nanoparticles and Al_2_O_3_-CEO nanoparticles, inhibition was observed for the *Escherichia coli* bacteria. Upon expanding the scale, the dilutions showing this inhibition were plated for colony counting (Figure 8). The concentration that achieved the highest inhibition was 50 mg/mL for Al_2_O_3_-CEO nanoparticles, resulting in a minimum inhibitory concentration of 7.80 × 10^7^ CFU/mL. For the *Enterococcus faecalis 29* bacteria, they did not have a strong inhibitory effect, suggesting that this approach might be more effective against other Gram-negative bacteria. This could be attributed to the functional group cinnamaldehyde in CEO, which acts as a growth inhibitor of *E. coli* but does not disintegrate the outer membrane or deplete intracellular ATP, implying a bacteriostatic effect [48]. Carrol et al. [49] reported that pure Al_2_O_3_ exhibited an inhibition zone of 8 mm in the disc diffusion test against *E. coli*, corresponding to a MIC of 500 µg/mL. For Gram-positive bacteria like *S. aureus*, the inhibition zone was 9 mm, indicating that Al_2_O_3_ has higher sensitivity to inhibit Gram-negative bacteria, aligning with the results of the present research. This could be because these bacteria, having two lipid membranes in their cell wall, interact with the lipid nature of the essential oil, causing damage to the cell walls and allowing the release of nutrients until the bacteria die. The literature indicates that cinnamon essential oil is sensitive to certain bacteria such as *Acinetobacter, Klebsiella pneumoniae*, *Proteus vulgaris*, *Enterococcus faecalis*, *Staphylococcus aureus*, and *Staphylococcus epidermidis*, with a reported MIC of 8 mg/mL, 2 mg/mL, 8 mg/mL, 4 mg/mL, 0. 5 mg/mL, and 1 mg/mL, respectively [50,51].

### 3.2. Characterization of Films

#### 3.2.1. FTIR

**Figure 9:**
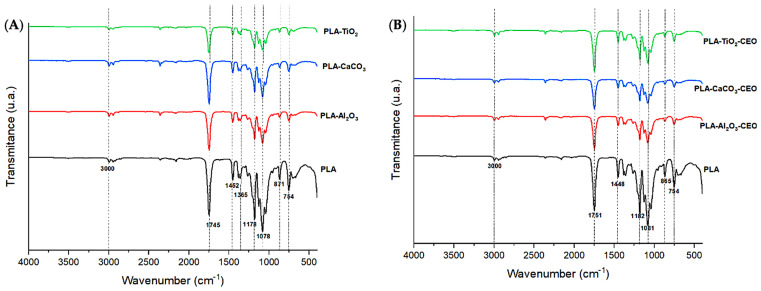
FTIR of PLA films with (**A**) pure nanoparticles and (**B**) nanoparticles modified with CEO.

FTIR analysis of the films was conducted to examine the infrared spectrum of the polymer and any potential alterations or modifications when adding metal oxide nanoparticles and those modified with CEO. In Figure 9A, spectra of PLA with pure nanoparticles (i.e., unmodified) are presented. PLA exhibits the following bands: 3000 cm^−1^, corresponding to the stretching vibration mode of the CH_3_ unsaturated hydrocarbon bond; a highly polar intense band at 1745 cm^−1^, referencing the C=O bond and its stretching vibration mode. Additionally, absorption bands at 1452 and 1365 cm^−1^ are observed, corresponding to the asymmetric and symmetric stretching vibration modes of the CH_3_ group. Other intense bands can be seen at wavelengths of 1178 and 1078 cm^−1^, attributed to the symmetric and asymmetric stretching vibration modes of the -C-O group, respectively. Finally, bands at around 871 and 754 cm^−1^ are found, corresponding to the amorphous and crystalline phases of PLA. These peaks in PLA correspond to those observed by other researchers [52,53]. It is noticeable that upon adding metal oxide nanoparticles to the polymer matrix, the characteristic bands decrease in intensity, suggesting that these nanoparticles have reduced the polarity of the interactions. Additionally, a slight shift of the 1745 cm^−1^ band to a higher wavenumber indicates that interactions with the nanoparticles have reduced the molecular weight of the polymer chains [54,55].

In Figure 9B, we observe the spectra of PLA with nanoparticles loaded with CEO. The bands described for pure PLA remain and there are no observable modifications among them. However, the intensity of the bands slightly decreases. As mentioned earlier, this effect may be due to low polarity. Thus, adding these nanoparticles modified with CEO does not affect the molecular structure of pure PLA. Famil et al. [56] examined a similar behavior in their PLA films with added SiO_2_ particles at different concentrations (1%, 3%, and 5%). When evaluating the FTIR spectrum, they found no changes in the molecular structure of PLA. Similarly, Heydari-Majd et al. [57] developed PLA composites with ZnO nanoparticles and essential oils of Zataria multiflora and Mentha piperita. They concluded that ZnO reinforcement did not modify any peak in PLA, except for a considerable reduction in the intensity of the peak at 3450 cm^−1^. In contrast, composites containing essential oils slightly decreased the intensity of the peaks. Qin et al. [58] reported that the addition of essential oils (tea tree, bergamot, lemongrass, rosemary, and clove) to the PLA matrix only caused changes in the intensity of the peaks, and it did not modify the molecular structure but did alter the intermolecular interactions.

#### 3.2.2. XRD

**Figure 10:**
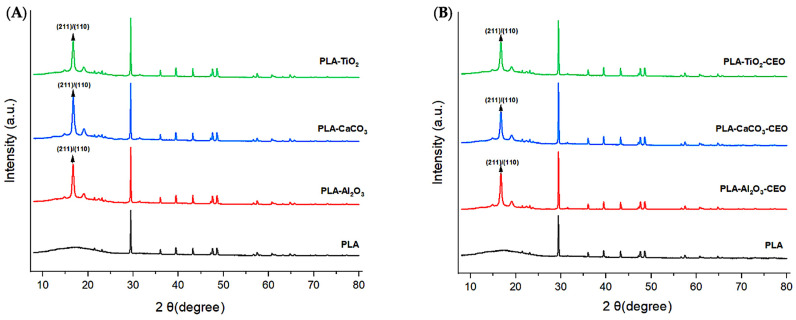
X-ray diffraction patterns in PLA films with (**A**) pure nanoparticles and (**B**) nanoparticles modified with CEO.

X-ray diffraction analysis is a quantitative and qualitative technique that identifies the phases present in a crystalline material and obtains information about its crystallographic properties. In this context, we obtained X-ray diffractograms to identify possible modifications in the PLA structure when adding metal oxide nanoparticles. Figure 10A depicts the obtained diffractograms of the films with each studied nanoparticle.

The pure PLA film exhibits a crystalline polymeric structure, showing characteristic peaks of this phase at 2*θ* values of 29.43°, 36.01°, 39.44°, 43.21°, 47.61°, and 48.67°. These characteristic peaks have been observed by other authors [59,60]. On the other hand, we can notice that adding Al_2_O_3_, CaCO_3_, and TiO_2_ nanoparticles tends to increase the order of the polymer chains due to the appearance of peaks at 16.69° and 19.21°. This increase in crystallinity corresponds to the characteristic crystallographic planes (200) and/or (110) of orthorhombic PLA crystal [61,62,63].

Figure 10B shows the diffractograms obtained for the PLA samples with nanoparticles loaded with CEO. Based on these diffractograms, the CEO does not generate alterations or modifications in the crystalline structure of pure PLA. However, the addition of nanoparticles as nanofillers can result in observable alterations. For instance, Yan et al. [64] reported that PLA films with added TiO_2_ nanoparticles at various concentrations (5%, 10%, and 20%) exhibited a significant increase in the peak at 25° in 2***θ***, indicating that the addition of metal oxides enhances the crystallinity of PLA. Chu et al. [65] indicated a similar effect when incorporating ZnO nanoparticles into PLA films, resulting in improved crystallinity compared to pure PLA. Mallick et al. [66] reached a similar conclusion, noting that the addition of TiO_2_ nanoparticles to PLA films improved both crystallinity and mechanical properties.

#### 3.2.3. SEM Analysis

**Figure 11:**
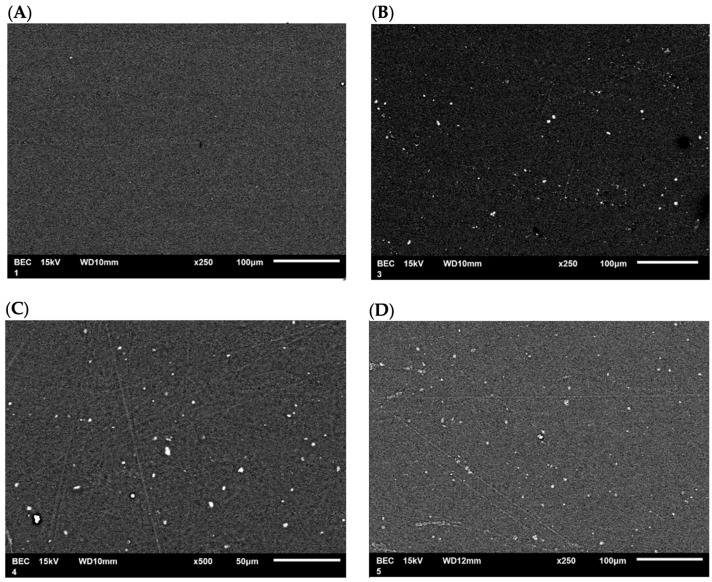
SEM micrographs in PLA films with (**A**) pure PLA, (**B**) TiO_2_-CEO, (**C**) CaCO_3_-CEO, and (**D**) Al_2_O_3_-CEO.

SEM micrographs of the films modified with CEO were obtained to observe the surface morphology of the PLA films and examine the distribution of the nanoparticles. The SEM micrograph (Figure 11A) of pure PLA shows a smooth film, indicating that the polymer was uniformly dissolved in the solvent. The SEM micrograph (Figure 11B) of the PLA film containing TiO_2_-CEO depicts the distribution of nanoparticles within the polymer network. The PLA film containing CaCO_3_-CEO exhibits a similar distribution of nanoparticles, as shown in Figure 11C. Also, the PLA film containing Al_2_O_3_-CEO displays nanoparticles distributed throughout the film, as illustrated in Figure 11D. Some small agglomerations can be observed due to static charges generated during the interaction of nanoparticles. However, most of the nanoparticles show a uniform distribution. Additionally, some line marks are visible on the films, likely resulting from the drying process in Petri dishes, which left their marks on the films. However, no scratches or ruptures are observed, indicating that the films were manufactured appropriately.

In the micrographs presented by Famil et al. [56], SiO_2_ nanoparticles in the films are uniformly distributed, displaying white and spherical dots on the PLA surface like our observations. Similarly, Shankar et al. [67] examined the morphology of their pure PLA films, registering a smooth and compact surface. In contrast, PLA films containing ZnO nanoparticles exhibited a constant and uniformly slightly rough distribution that increased with higher nanoparticle concentrations. Qin et al. [58] developed PLA films without nanoparticles but with the addition of essential oils such as rosemary, lemongrass, bergamot, and clove, reporting that the film morphology showed cavities and pores due to the lipidic nature of the essential oils. This lipidic nature contributed to lower tensile strength and higher permeability of the films.

#### 3.2.4. DSC Analysis

**Figure 12:**
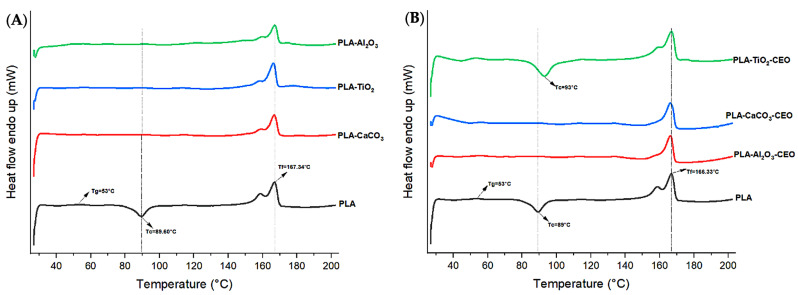
Thermal DSC curves in PLA films with (**A**) pure nanoparticles and (**B**) nanoparticles modified with CEO.

In Figure 12, we observe the curves obtained from differential scanning calorimetry (DSC) for films with CEO-modified nanoparticles and unmodified ones. In Figure 12A, the pure PLA film exhibits a glass transition temperature (Tg) at 53 °C, indicating that the material starts changing its physical state and becomes softer at this temperature. At 89 °C, there is an exothermic peak, suggesting slow crystallization during the heating scan, which precedes its crystallization temperature before reaching the melting temperature of 167.34 °C. Unlike the films with TiO_2_, Al_2_O_3_, and CaCO_3_ nanoparticles, there is no observable cold crystallization during the heating scan in the pure PLA film. This could be because the nanoparticles improved the crystallization of pure PLA, as they rapidly crystallized during their addition. In Figure 12B, the curves for films with Al_2_O_3_-CEO and CaCO_3_-CEO-modified nanoparticles show similar effects, with no observable cold crystallization during the heating scan. However, the film containing TiO_2_-CEO nanoparticles exhibits a curve very similar to that of pure PLA, with a slight 4 °C shift in Tg, possibly due to the resistance offered by TiO_2_ to pure PLA. Both transition temperatures (Tm and Tg) are like those of pure PLA in both thermal transition curves.

Table 2 summarizes the thermal properties (fusion enthalpy and degree of crystallization) obtained from the DSC curve. The film containing Al_2_O_3_-CEO nanoparticles presented the highest degree of crystallization at 42%, translating to a 130.36% increase compared to pure PLA. Similar results were reported by Heidary-Majd et al. [57], in which pure PLA showed a Tg of 54 °C. However, films containing ZnO nanoparticles with essential oils *Zataria multiflora* and *Mentha piperita* exhibited a range from 45 to 56.5 °C for Tg, indicating that essential oils reduced the glass transition temperature by 16.66%. The reported Tm was in the range from 147 to 131 °C, lower than that observed in the present research, and crystallinity percentages were not provided. Shankar et al. [67] added ZnO nanoparticles at concentrations of 5%, 1%, and 1.5% to the PLA matrix, maintained Tg (54.3 °C), Tc (99.7 °C), and Tm (167.7 °C) in all samples. They observed a variation in the percentage of crystallinity for PLA, with an increase of 12.7% and a 60.62% increase at the 1.5% nanoparticle concentration. However, this increase was less than that obtained by adding Al_2_O_3_-CEO, suggesting that the interaction between these components significantly improved PLA crystallinity. In contrast, Qin et al. [58] incorporated essential oils to PLA films and reported Tg (55 °C), Tc (108.4 °C), Tm (166.7 °C), and Xc (11.7%) for pure PLA. These values were slightly reduced with the addition of essential oils. However, an increase in the percentage of crystallinity of 41.88% was observed, especially with the addition of clove essential oil. This emphasizes the potential benefits of combining essential oils in such studies.

#### 3.2.5. Water Vapor Permeability

**Figure 13:**
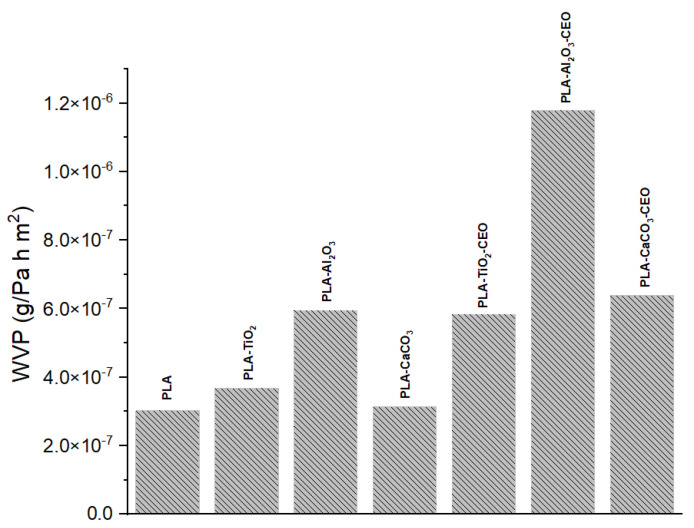
Water vapor permeability results of PLA films with pure nanoparticles and nanoparticles modified with CEO.

Water vapor permeability is one of the most crucial characteristics when working with films intended for packaging use. It is a barrier property that allows us to understand how quickly water vapor can transfer. Figure 13 shows the results of water vapor permeability in PLA films incorporating pure nanoparticles with and without CEO modification. The pure PLA film maintains the lowest permeability (3.02 × 10^−7^ g/Pa·h·m^2^), indicating that the arrangement of the polymeric chains hinders the transfer of water vapor. On the other hand, films with TiO_2_ and CaCO_3_ nanoparticles showed similar permeability to pure PLA, with values of 3.67 × 10^−7^ g/Pa·h·m^2^ and 3.13 × 10^−7^ g/Pa·h·m^2^, respectively. However, the PLA film with Al_2_O_3_ nanoparticles exhibited higher permeability at 5.94 × 10^−7^ g/Pa·h·m^2^, suggesting that these nanoparticles create open spaces between the chains through which water vapor can easily flow. 

Furthermore, PLA films containing nanoparticles loaded with CEO showed a significant increase in their permeability. This effect could be attributed to the drying process of the PLA film, where the solvent can evaporate at room temperature. Some CEO molecules may evaporate with the solvent, producing open spaces where water vapor transfer is not strongly impeded. The incorporation of different essential oils such as pimpinella anisum [68], zataria multiflora [69], oregano [70], zataria multiflora and peppermint [57], and limonene [71] could increase the porosity of PLA films, which facilitates the water vapor transfer. For instance, Chu et al. [65] reported a PLA permeability of 1.69 × 10^−14^ kgm/(m^2^sPa), which increased by 93% with the addition of ZnO nanoparticles. This improvement was attributed to enhanced hydrophilic interactions within the films. Instead, Yakdoumi et al. [72] found that the addition of nanofillers, such as TiO_2_ and Al_2_O_3_, without essential oils reduced the water vapor permeability (WVP) in the composites. The WVP reduction was observed as follows: PLA-TiO_2_ (47%), PLA-Al_2_O_3_ (39%), and PLA-TiO_2_-Al_2_O_3_ (54%). This suggests that the addition of metal oxides in PLA films can influence this property based on interactions between the components. This analysis can be useful as an indication of potential applications for these films.

### 3.3. Mechanical Properties

**Figure 14:**
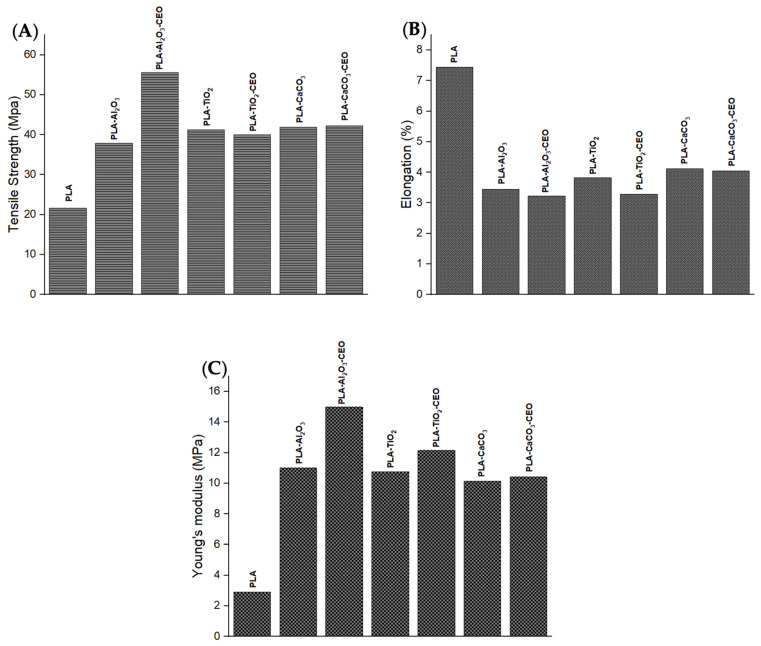
Mechanical properties of PLA films with pure nanoparticles and nanoparticles modified with CEO: (**A**) tensile strength, (**B**) elongation, and (**C**) Young’s modulus.

The assessment of the mechanical properties of the materials provides useful information for their potential applications and limitations. Therefore, we examined the behavior of films made with metal oxide nanoparticles and films loaded with cinnamon essential oil (CEO). Figure 14 depicts the properties of the PLA films with pure nanoparticles and nanoparticles modified with CEO, such as tensile strength, elongation, and Young’s modulus.

Tensile strength measures the resistance of a material to breaking when subjected to a force. Pure PLA films exhibited lower tensile strength (21 MPa) than films containing nanoparticles, as shown in Figure 14A. Thus, the PLA films with nanoparticles are stronger, increasing their tensile strength by around 50% compared to pure PLA films. The PLA film containing Al_2_O_3_ nanoparticles modified with CEO has the highest tensile strength. This could be attributed to good interaction between the nanoparticles and the PLA polymer matrix, increasing intermolecular forces between the chains. 

Elongation represents the percentage by which a material can stretch before breaking. Pure PLA film registers the highest elongation, indicating greater chain mobility with a 7% elongation, as shown in Figure 14B. PLA films containing nanoparticles have elongation values in the range of 3% to 4%, and there are no significant differences with PLA films containing CEO. This suggests that the CEO did not directly affect this property.

Young’s modulus refers to the resistance of a material to elastic deformation when subjected to force. Pure PLA film has the lowest Young’s modulus. However, PLA films containing nanoparticles both with and without CEO significantly increase their Young’s modulus (Figure 14C). These films have good mechanical strength when subjected to tensile or compressive forces. These films could be useful for active packaging applications for transportation due to their better mechanical strength. It could increase the mechanical performance and lifetime of the products that contain these films [73,74]. Famil et al. [56] reported that the incorporation of SiO_2_ nanoparticles increased the crystallization of PLA and improved its mechanical properties. The tensile strength of the PLA film increased to 10.34%, reaching a maximum of 48.27% with a concentration of 5% SiO_2_ compared to pure PLA. Additionally, this PLA film increased its Young’s modulus to 34.78% and decreased its elongation to 66%. Also, Shankar et al. [67] assessed better mechanical properties of the PLA film by adding ZnO nanoparticles. Their results showed that PLA films with 1.5% of ZnO nanoparticles improved their tensile strength by 52.9 MPa, elongation by 4.4%, and Young’s modulus by 2.44 GPa.

In contrast, Qin et al. [58] indicated that the addition of essential oils to PLA films decreased their mechanical properties. For instance, by incorporating clove essential oil, the tensile strength and Young’s modulus of the PLA film decreased by 169% and 137.48%, respectively, while its percentage of elongation was increased by 200%. Therefore, combining essential oils and metal oxide nanoparticles can be used in PLA films to modify their mechanical properties.

## 4. Conclusions

We investigated the variations in some properties of pure nanoparticles of TiO_2_, CaCO_3_, and Al_2_O_3_ when modified with cinnamon essential oil. For instance, their infrared spectrum and thermogravimetric analysis. TiO_2_ and CaCO_3_ nanoparticles did not efficiently capture terpenoids on their surfaces, resulting in no inhibitory effects on microorganisms and low antioxidant capacity. On the other hand, Al_2_O_3_ nanoparticles registered greater interaction efficiency with CEO, showing a 40% increase in antioxidant capacity compared to pure Al_2_O_3_ nanoparticles. Also, these nanoparticles transferred antimicrobial properties for inhibiting Gram-negative bacteria at a minimal nanoparticle concentration of 50 mg/mL.

The addition of nanoparticles of TiO_2_, CaCO_3_, and Al_2_O_3_, both pure and modified with CEO, caused significant changes in the properties of PLA film. These changes improved the mechanical and thermal properties of the PLA film. The water vapor permeability properties of the PLA film indicated an increase due to the release of spaces during film drying. The films exhibited good morphology, with smooth surfaces and even nanoparticle distribution within the polymer network. In these results, we did not observe scratches or fissures.

Pure or CEO-modified nanoparticles can be used in some polymers to improve their tensile strength and Young’s modulus. Also, these nanoparticles can be employed in the active packaging of products to improve their lifetime and performance. Active packaging can incorporate antioxidant and antimicrobial particles, effectively retarding food spoilage. This innovation is an alternative to the synthetic preservatives that have undesirable health effects on consumers. In recent years, active packaging technology has significantly grown, increasing the investigations to improve the packaging properties. However, not all metal oxides can alter their properties using essential oils due to factors such as molecular structure, availability of active sites, nanoparticle size, and polarity. For future research, we will explore new alternatives or methodologies to address these challenges.

## Figures and Tables

**Table 1:** Results of the drop plate method with nanoparticles at different concentrations.

Sample	Bacteria		Concentrations
	*E. coli*	*E. faecalis 29*	
CaCO_3_	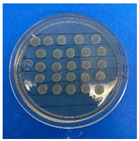	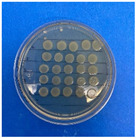	+ 200 mg/mL 100 mg/mL 50 mg/mL 25 mg/mL −
CaCO_3_-CEO	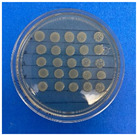	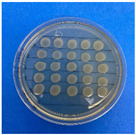	+ 200 mg/mL 100 mg/mL 50 mg/mL 25 mg/mL −
TiO_2_	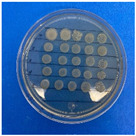	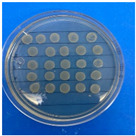	+ 200 mg/mL 100 mg/mL 50 mg/mL 25 mg/mL −
TiO_2_-CEO	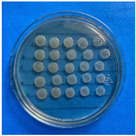	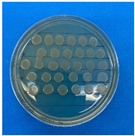	+ 200 mg/mL 100 mg/mL 50 mg/mL 25 mg/mL −
Al_2_O_3_	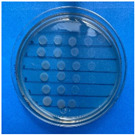	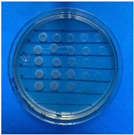	+ 200 mg/mL 100 mg/mL 50 mg/mL 25 mg/mL −
Al_2_O_3_-CEO	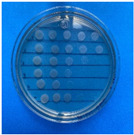	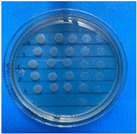	+ 200 mg/mL 100 mg/mL 50 mg/mL 25 mg/mL −

**Table 2:** Thermal properties of the pure PLA films with pure nanoparticles and nanoparticles modified with CEO.

Sample		Cold Crystallization		Melting		Crystallinity
	Tg (°C)	Tc (°C)	${∆H}_{m}$ (J/g)	Tm (°C)	${∆H}_{c}$ (J/g)	Xc (%)
PLA	53	89.51	22.78	166.79	40.89	18.61
PLA-TiO_2_-CEO	56	93	39.23	166.09	46.82	7.80
PLA-CaCO_3_-CEO	57	-	-	166.56	18.93	19.45
PLA-Al_2_O_3_-CEO	56	-	-	166.32	41.72	42.87
PLA-TiO_2_		-	-	166.61	37.21	38.24
PLA-CaCO_3_		-	-	166.90	26.53	27.27
PLA-Al_2_O_3_		-	-	167.47	39.24	40.33

## Data Availability

Data is contained within the article.
